# Co-localization of and interaction between duck enteritis virus glycoprotein H and L

**DOI:** 10.1186/s12917-018-1553-6

**Published:** 2018-08-29

**Authors:** Daishen Feng, Min Cui, Renyong Jia, Siyang Liu, Mingshu Wang, Dekang Zhu, Shun Chen, Mafeng Liu, Xinxin Zhao, Yin Wu, Qiao Yang, Zhongqiong Yin, Anchun Cheng

**Affiliations:** 1 Research Center of Avian Disease, College of Veterinary Medicine, Sichuan Agricultural University, Chengdu, 611130 Sichuan China; 2 Institute of Preventive Veterinary Medicine, Sichuan Agricultural University, Chengdu, 611130 Sichuan China; 3 Key Laboratory of Animal Disease and Human Health of Sichuan Province, Chengdu, 611130 Sichuan China

**Keywords:** DEV, Glycoprotein H, Glycoprotein L, Interaction, Co-localization, Co-immunoprecipitation

## Abstract

**Background:**

Duck Enteritis Virus (DEV), belonging to the α-herpesvirus subfamily, is a linear double-stranded DNA virus. Glycoprotein H and L (gH and gL), encoded by UL22 and UL1, are conserved in the family of herpesviruses. They play important roles as gH/gL dimers during viral entry into host cells through cell-cell fusion. The interaction between gH and gL has been confirmed in several human herpesviruses, such as Herpes Simplex Virus (HSV), Epstein-Barr virus (EBV) and Human Cytomegalovirus (HCMV). In this paper, we studied the interaction between DEV gH and gL.

**Results:**

Recombinant plasmids pEGFP-N-gH and pDsRED-N-gL were constructed successfully. Expressions of both DEV gH and gL were observed after incubation of COS-7 cells transfected with pEGFP-N-gH and pDsRED-N-gL plasmids after 12 h, respectively. Also, the co-localization of a proportion of the gH and gL was detected in the cytoplasm of COS-7 cells after co-transfection for 24 h. Then, pCMV-Flag-gL and pCMV-Myc-gH recombinant plasmids were constructed and co-transfected into COS-7 cells. It was showed that both gH and gL were tested with positive results through co-immunoprecipitation and Western-blotting.

**Conclusions:**

Our results demonstrated not only the co-localization of DEV gH and gL in COS-7 cells, but also the interaction between them. It will provide an insight for the further studies in terms of protein-protein interaction in DEV.

## Background

Duck Enteritis Virus (DEV) is a linear double-stranded DNA virus, of the α-herpesvirus subfamily [34]. Herpesviruses, consisting of core, capsid, tegument and envelope, are a large family of the linear double-stranded DNA group of viruses [27, 28]. It has three subfamilies including α-, β- and γ- herpesvirus. The α-herpesvirus contains: Herpes Simplex Virus 1 (HSV-1), Herpes Simplex Virus 2 (HSV-2), Varicella-zoster Virus (VZV), and Pseudorabies Virus (PRV); β-herpesvirus: Human Cytomegalovirus (HCMV), Human Herpesvirus 6 and 7 (HHV-6 and HHV-7); and γ-herpesvirus: Epstein-Barr virus (EBV) and Kaposi’s sarcoma-associated herpesvirus (KSHV, also known as Human Herpesvirus 8 (HHV-8)) [5, 19, 32]. DEV, also known as Duck Plague Virus (DPV) and Anatid Herpesvirus 1 (AnHV-1), mainly causes acute and contagious infection to waterfowl. Until now, the duck enteritis virus is one of the most severe diseases that seriously affect economy in the worldwide duck industry [23, 39].

Glycoprotein H is highly conserved in the herpesviruses. Heterodimer gH/gL, commonly formed through non-covalent linkages, is of great importance to membrane fusion of the enveloped viruses and host cells [6]. It has been studied that in the process of HSV-1 and HCMV entry into host cells, gB, gH and gL are three essential glycoproteins for the cell-cell fusion [1, 2, 24, 26]. Firstly, glycoproteins of herpesviruse bind with their specifically cellular receptors, in order to attaching to the membrane of host cells [10]. Then, gB, as a fusogen, is activated by gH/gL through interaction, therefore the fusogenic function of gB is promoted [23, 24, 30]. The function of connection between gB and gH/gL, also known as “core fusion machinery,” is essential for herpesviruses to initiate membrane fusion [15]. For example, the first step of EBV entry into Epithelial cells is tethering gH/gL to its cellular membrane receptors, including integrin αγβ5, αγβ6 and αγβ8, which will change the conformation of gH/gL delicately due to receptor-binding. Then, the heterodimer will interact with gB and trigger its fusogenic function [5, 10, 11]. Therefore, gH/gL is more likely a regulator in the process of cellular membrane fusion.

Recently, the three-dimensional crystal structures and interaction domains of gH/gL of HSV-2 and EBV gH/gL have been solved [8, 26], which helped immensely in understanding the structure and function of gH/gL. However, the functional mechanisms of gH/gL are complicated and diverse in different herpesviruses. For instance, although the structural conformation of gH/gL is generally homologous among HSV-2, EBV and HCMV, some differences still exist, including divided domains, interdomain packing angles and fragment antigen-binding regions of the neutralizing antibody [8, 10]. When gH binds gL at the N-terminal domain (H1) of HSV-2, gH/gL therefore forms a boot-like heterodimer with a ∼ 60° kink between domain H2 and H3 at the C terminus [8]. Similarly, comprising a kink between domains III and IV, the structure of HCMV gH/gL has the semblable boot-like heterodimer as HSV-2 [8–10, 26]. Furthermore, in most human herpesviruses, gL requires correct enfoldment to bind with gH. HSV-1 gL is more likely a scaffolding protein than a chaperone to traffic gH and promotes its surface expression, then gH anchors gL to the cell surface [12, 20, 33]. During membrane fusion, the fusogenic potential of HSV-1 gB is activated through interaction with gH/gL, before the alteration of gH/gL is initiated by gD [1, 4]. In short, the main function of HSV gH/gL is activating gB through direct interaction during entry of host cells.

Although the structure and function of gH/gL in some human herpesviruses have been studied to some extent, for example the interaction regions of gH and gL of HSV-2 and EBV have been studied and published already [8, 26], there is insignificant research data in the study of DEV gH and gL until now. According to the existence of gH/gL dimers in other herpesviruses, the hypothesis that DEV gH would interact with gL was proposed in this paper. In this study, the two expression plasmids, pEGFP-N-gH and pDsRED-N-gL, were co-transfected into COS-7 cells to analyze the co-localization of DEV gH and gL. Then, the co-immunoprecipitation (Co-IP) was conducted in order to detect the interactions between these two proteins. As a result, our study provided an evidence of interaction between DEV gH and gL, which will be helpful for the further study about the protein-protein interaction in DEV as well as other herpesviruses.

## Methods

### Virus and DNA extraction

The DEV CHv strain (GenBank No. JQ647509.1) was precured from Avian Disease Research Center of Sichuan Agricultural University. Monolayer cultures of duck embryo fibroblasts (DEFs) were infected with DEV CHv strain at a multiplicity of infection (MOI) of 0.1, which was incubated in Dulbecco’s modified Eagle medium (DMEM) containing 10% fetal bovine serum (FBS). The use of duck embryos was approved by the Animal Ethics Committee of Sichuan Agricultural University (approval No. XF2014–18). Then, the cells were incubated at 37 °C until a cytopathic effect (CPE) appeared in about 80% of the cells. DNA of DEV was extracted from the infected cell lysis using DNA Extraction Kit for Virus (TianGen Biotech Co., LTD.).

### Primer design and PCR amplification

A bioinformatics analysis of DEV CHv genome sequence (GenBank No. JQ647509.1) was performed and a pair of primers was designed to amplify the truncate UL1 gene (UL1t). The forward primer UL1t-1F and reverse primer UL1t-1R were showed in Table 1 (the underlined sequences was restriction enzyme *Hind*III site).

**Table 1:** The primers of amplification for both DEV UL1 and UL22

Name of Primers	Primers	Restriction Enzyme
UL1t-F	5′- CCGGAATTCATGTACCCGGTTATCGAAGGAG − 3′	*EcoR*I
UL1t-R	5′- CCCAAGCTTTCGTCGGCCATGGTGTCCGGG − 3	*Hind*III
UL1-1F	5’-CGGAATTCATGGGTTCCAGATGGAAGGC-3′	*EcoR*I
UL1-1R	5′- GGGGTACCCCTCGTCGGCCATGGTGTCCGG − 3′	*Kpn*I
UL1-2F	5′- CCGGAATTCATGGGTTCCAGATGGAAGGCC -3′	*EcoR*I
UL1-2R	5’-ACGCGTCGACCTATCGTCGGCCATGGTGTCC -3′	*Sal*I
UL22-1F	5′- CGGAATTCATGTCGCAGCTTACGGTGC -3’	*EcoR*I
UL22-1R	5′- GGGGTACCCCTTCTTCATTGCTTAACAGTTC -3’	*Kpn*I
UL22-2F	5′- CGGAATTCATGTCGCAGCTTACGGTG −3’	*EcoR*I
UL22-2R	5′- CCCTCGAGTCATTCTTCATTGCTTAACAG − 3’	*Xho*I

Two pairs of primer were designed to amplify the UL1 and UL22 gene respectively, which encode the gL and gH of DEV respectively. The two pairs of primer for amplifying UL1 gene are seen in Table 1 as UL1-1F/1R and UL1-2F/2R respectively. The other two pairs of primer for amplifying UL22 gene are also shown in Table 1 as UL22-1F/1R and UL22-2F/2R respectively. The DEV CHv DNA was used as template. The PCR was performed with 10 μL mixture as reactions containing 5 μL PrimeSTAR (premix) DNA polymerase, 0.2 μL each of primer at the concentration of around 5 nmol/OD, 0.4 μL DNA template at the concentration of 280 ng/μL, and 4.2 μL H_2_O. Then, the PCR amplification was performed in pre-denaturation at 98 °C for 2 min, denaturation at 98 °C for 10s, annealing at 55 °C for 30s, extension at 72 °C for 30s, and final extension at 72 °C for 10 min after 30 cycle repeats.

### Plasmid construction

Expression vectors pET-32a(+), pEGFP-C, pDsRED-N, pCMV-Flag and pCMV-Myc were available in the laboratory. After digested with both *EcoR*I and *Hind*III restriction enzymes respectively, the truncate DEV UL1 gene and vector pET-32a(+) were both linked to the recombinant expression plasmid pET-32a(+)-UL1t with solution I ligase. Plasmids pEGFP-N-gH and pDsRED-N-gL were constructed in the same way as above by digestion with *EcoR*I and *Kpn*I, while the plasmid pCMV-Flag-gL was constructed by digestion with *EcoR*I and *Sal*I, and pCMV-Myc-gH with *EcoR*I and *Xho*I*.* Once these plasmids were constructed successfully, they were identified by restriction enzyme digestion and nucleotide sequencing.

### Antibodies

Mouse polyclonal antibody against DEV CHv gL was prepared with purified UL1t protein, and identified by both indirect immunofluorescence and western-blotting respectively, which was detailed in the next section. The anti-Flag mouse and anti-Myc rabbit polyclonal antibodies (Beyotime Institute of Biotechnology, China) were available in the laboratory.

### Cells culture and transfection

COS-7 cell lines, available in the laboratory, were cultured in DMEM supplement with 10% FBS at 37 °C in 5% CO_2_ incubator. Mixture of transfection reagent containing recombinant plasmids, DMEM, Lipofectainine 3000 and P3000 (Invitrogen, USA) were used to transfect COS-7 cells with 6-well plates. 250 μL of the transfection reagent was seeded into each well of 6-well plates containing COS-7 cells at densities of 78–80%. DMEM was supplemented in each well up to 2 mL with 2% FBS. Then, the cells were cultured at 37 °C in 5% CO_2_ incubator.

### Preparation of DEV CHv anti-gL mouse polyclonal antibody

Recombinant plasmid pET-32a(+)-UL1t was transformed into *E. coli* Rosetta and induced to expression for 2 h, 3 h, 4 h and 5 h with 1 mM IPTG at 37 °C to ensure the best induced time, and induced to expression with 0.2, 0.4, 0.6, 0.8, 1.0 mM IPTG at 37 °C for 4 h to ensure the best concentration of IPTG. Then, UL1t protein was induced with 0.6 mL IPTG at 37 °C for 4 h following purified with nickel agarose to bind with the His-tag of the pET-32a(+)-UL1t plasmid. Finally, the purified UL1 protein was confirmed by SDS-PAGE analysis (data not shown). Four male Kunming mice (Chengdu Dashuo Laboratory Animal Technology Co., Ltd) around 18-22 g of 6 weeks old were immunized with purified UL1t protein for four times at 4r week intervals. After immunization, the serum containing anti-gL polyclonal antibody was collected, and the euthanasia via cervical dislocation of mice was performed according to the Canadian Council on Animal Care (CCAC) guidelines and the American Veterinary Medical Association (AVMA) guidelines.

### Indirect immunofluorescence assay

After plasmids transfection for 48 h, the cells were processed as follows: fixation with 4% paraformaldehyde, permeabilization of cells with 0.25% Triton-X100, and sealing with 5% BSA for 1 h separately. The cells were incubated with one of the following antibodies for 1 h: anti-Myc rabbit or anti-gL mouse polyclonal antibody (1:1000 dilution), and incubated with goat anti-rabbit IgG or goat anti-mouse IgG secondary antibodies (1: 1000 dilution) for 1 h. Also, the cell nucleus was stained with 4′,6-diamidino-2-phenylindole (DAPI). Finally, cells were observed by fluorescence microscopy (Nikon, Japan) [31].

### Co-immunoprecipitation

Based on the protocol of Co-immunoprecipitation [14], the COS-7 cells were harvested in phosphate buffered saline (PBS) after co-transfection of experimental group (pCMV-Flag-gL and pCMV-Myc-gH recombinant plasmids), and control group (Control 1: pCMV-Flag-gL and pCMV-Myc or Control 2: pCMV-Flag and pCMV-Myc-gH plasmids) into COS-7 cells respectively for 48 h, followed by incubation in lysis buffer (containing 1% PMSF) for 30 min on ice. Then, the cells were centrifuged for 30 min in a 4 °C (Thermo Fisher Scientific, USA) and 14,000×g speed to pellet debris. SureBeads Magnectic Beads System (Bio-Red) was introduced in the Co-IP experiment. SureBeads Protein A was washed thrice in PBST and added 100 μL into every 200 μL IP antibody (anti-Myc rabbit or anti-gL mouse polyclonal antibody were used at dilutions of 1:1000). After incubation for 30 min at room temperature, the beads were pulled to the side of the tube by the Magnetic Racks, followed by discarding of the supernatant. After being washed three times with PBST, the beads were added into the lysates, and incubated at room temperature for 1 h. Finally, the beads were pulled to the side by the Racks, and washed three times with PBST, followed by mixing evenly with 1 × SDS loading buffer for analysis through Western blotting [2].

### Western blot

The extracts or lysates with loading buffer were electrophoresed by SDS-PAGE, and then electro-transferred to polyvinylidene fluoride (PVDF) membranes through trans-blot SD (Bio-Rad, USA). Membranes were incubated with one of the antibodies as follows: anti-gL mouse polyclonal antibody (1: 1000 dilution), anti-Flag mouse or anti-Myc rabbit polyclonal antibody (1: 1000 dilution). Then, either the HRP-conjugated goat anti-rabbit IgG or goat anti-mouse IgG (1: 3000 dilution) was incubated as secondary antibody. The membrane was washed three times by tris-buffered saline and tween 20 (TBST, containing 8 g NaCl, 0.2 g KCl, 3 g Tris-base, 0.05% Tween 20 in each 1 L, pH 7.4) after incubation with antibodies in each of the step above, and finally detected through ECL reagent [38].

## Results

### Construction of the expression plasmids

**Fig. 1:**
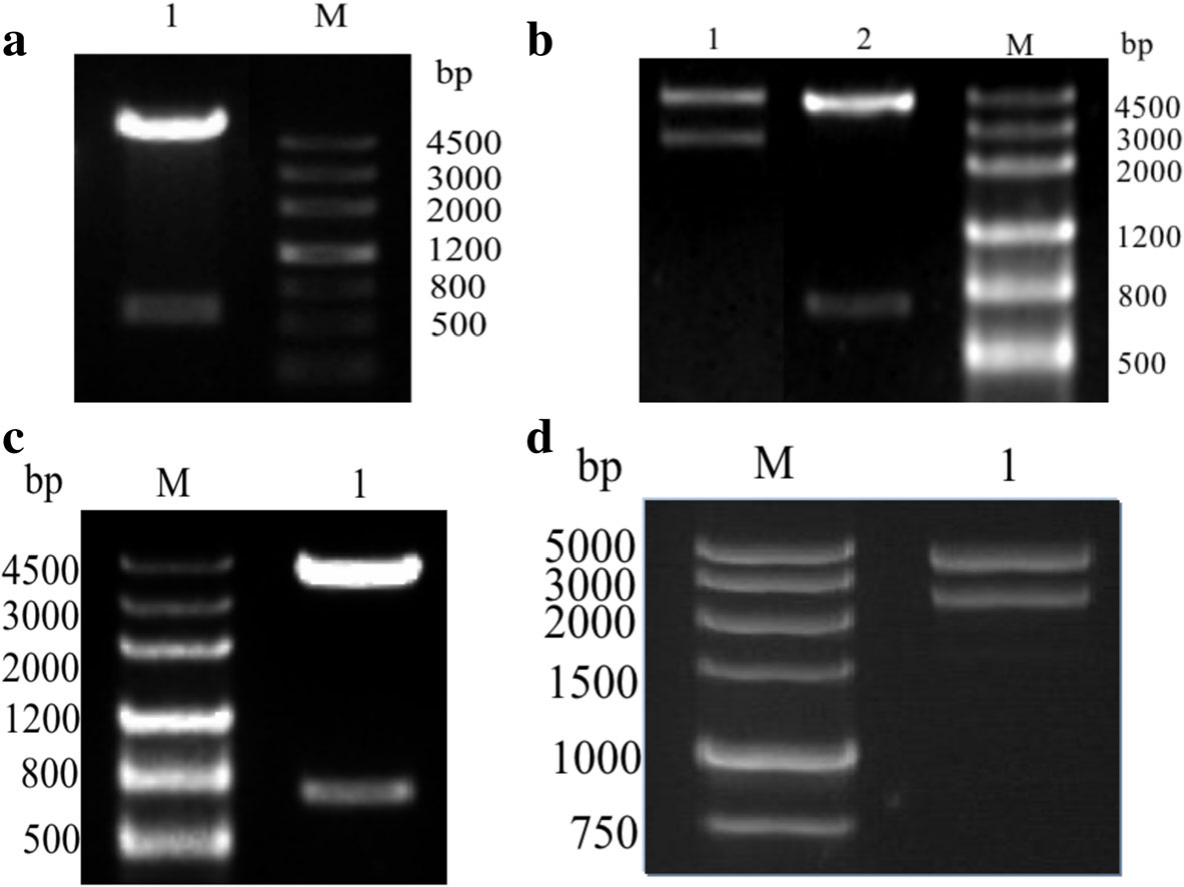
The identification of recombinant plasmids with enzyme digestion The expression plasmids were identified through enzyme digestion. **a** Lane 1: pET32a (+)-UL1t digested with two target enzymes; M: DNA Marker. **b** Lane 1: pEGFP-N-gH digested with two target enzymes; Lane 2: pDsRED-N-gL digested with two target enzymes; M: DNA Marker. **c** Lane 1: pCMV-Flag-gL digested with two target enzymes; M: DNA Marker. **d** Lane 1: pCMV-Myc-gH plasmid digested with two target enzymes; M: DNA Marker.

Sequencings of amplified DEV CHv UL1 and UL22 were identified through alignment with known sequences published in NCBI. The structure of recombinant plasmid pET-32a(+)-UL1t was confirmed by digestion with the restriction enzymes *EcoR*I and *Hind*III (Fig. 1a) and sequencing. The plasmids pEGFP-N-gH and pDsRED-N-gL were also identified by digestion with *EcoR*I and *Kpn*I (Fig. 1b), while the plasmid pCMV-Flag-gL was confirmed by digestion with *EcoR*I and *Sal*I (Fig. 1c), and pCMV-Myc-gH with *EcoR*I and *Xho*I (Fig. 1d)*.* These recombinant plasmids were further confirmed by sequencing analysis (data not shown).

### Intracellular localization of DEV gL and gH in COS-7 cells respectively

**Fig. 2:**
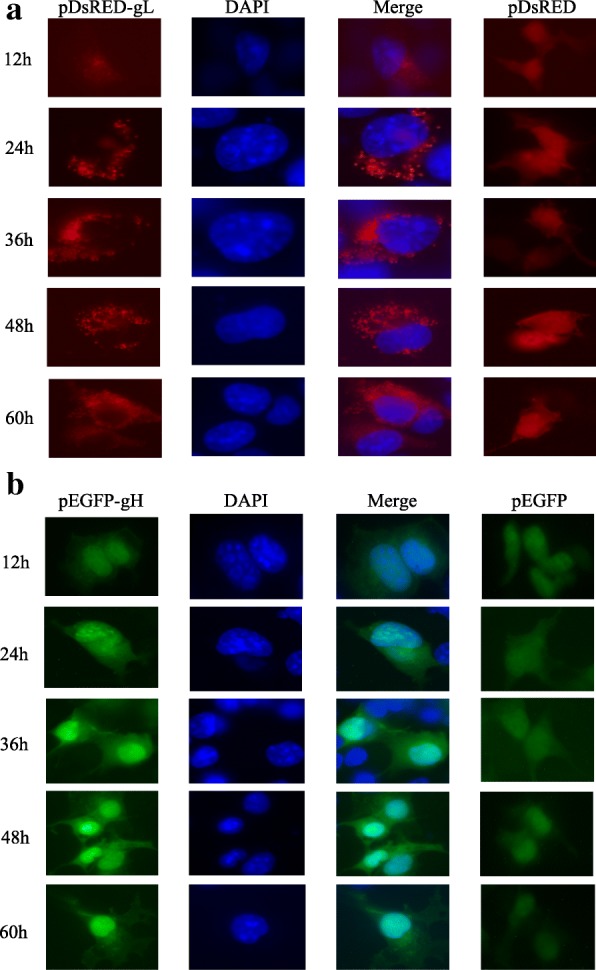
Intracellular localization and expression phase of DEV gH and gL respectively in COS-7 cells. **a** In the first column, red shows the expression and intracellular localization of pDsRED-N-gL. In the second column, blue shows the nuclear stained with DAPI. The third column represents the merged images. The vector pDsRED-N as a negative control was showed in the fourth column. **b** Similarly, green shows the expression and intracellular localization of pEGFP-N-gH; blue shows the nuclear stained with DAPI. The vector pEGFP-N as a negative control was showed in the fourth column

After transfection of COS-7 cells with pDsRED-N-gL and pEGFP-N-gH expression plasmids respectively, the cells were observed by fluorescence microscopy in 12 h, 24 h, 36 h, 48 h and 60 h. A red low fluorescence of plasmid pDsRED-N-gL was observed starting 12 h post transfection, with gradual increase in intensity from 24 h to 60 h, suggesting that the expression of gL started at 12 h and increased from 24 h to 60 h after transfection. The observation of red fluorescence also indicated the localization of expressed gL in COS-7. Compared with the even distribution in the cells of pDsRED (negative control), pDsRED-N-gL was randomly distributed as dots around the nucleus which was stained with DAPI in blue (Fig. 2a). Similarly, the observation of green fluorescence suggested the localization of expressed gH in COS-7. The green fluorescence of plasmid pEGFP-N-gH started at 12 h and increased from 24 h to 60 h after transfection. It also showed that pEGFP-N-gH was randomly distributed in the cytoplasm as well as the nucleus, while the EGFP was distributed in the entire cells at 12 h, 24 h, 36 h, 48 h and 60 h (Fig. 2b). Thus, the expression of both DEV gL and gH appears to have started at 12 h, and increased from 24 h to 60 h after transfection. Finally, gL localized in the cytoplasm while the gH localized in both of the cytoplasm and the nucleus in COS-7 cells.

### Co-localization of gH and gL in COS-7 cells

**Fig. 3:**
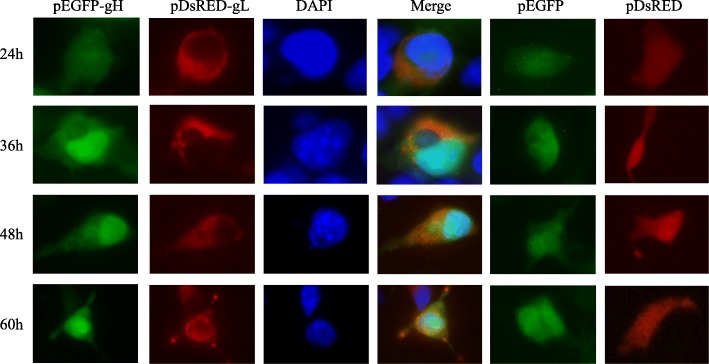
Co-localization of DEV gH and gL in COS-7 cells. In the first column, green shows the localization of pEGFP-N-gH. In the second column, red shows the localization of pDsRED-N-gL. In the third column, blue shows the nuclear stained with DAPI. The merged imagine of co-localization of gH and gL was showed in the fourth column. The vectors pEGFP-N and pDsRED-N were showed as negative controls in the fifth and sixth columns

In order to ascertain the co-localization of gH and gL, the two plasmids pDsRED-N-gL and pEGFP-N-gH were co-transfected into COS-7 cells. The vectors pDsRED-N and pEGFP-N were co-transfected into COS-7 cells as negative controls. After the nucleus was stained with DAPI, the cells with co-transfection plasmids were observed through fluorescence microscope (Fig. 3). As seen in Fig. 2, pDsRED-N-gL expressed as red fluorescence while pEGFP-N-gH expressed as green fluorescence. The expression was detected at 24 h, and gradually increased until 60 h. Interestingly, when the expression signals of pDsRED-N-gL, pEGFP-N-gH and nucleus merged together, optically an orange fluorescence appeared at points throughout the cytoplasm. If merged results suggest that the orange dots represented the co-localization sites of gH and gL (Fig. 3), then the fluorescence microscope observation as described above indicates the partial co-localization of gH and gL in COS-7 cells.

### Co-immunoprecipitation analysis of the interaction of gH and gL

**Fig. 4:**
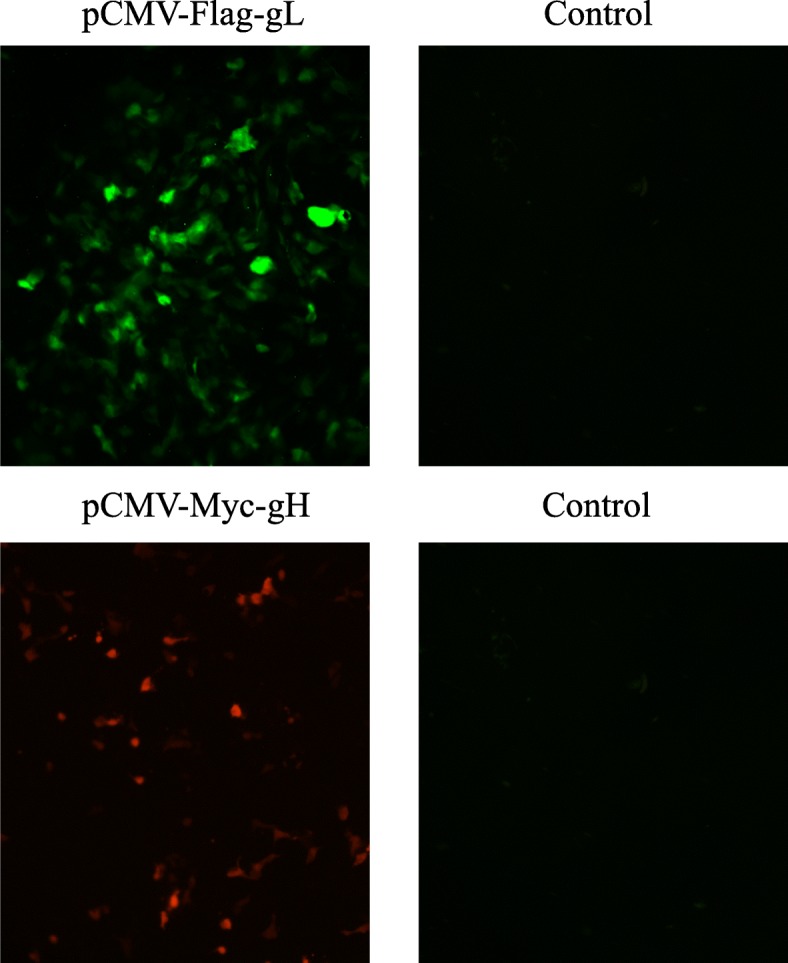
Identification of expressions of pCMV-Flag-gL and pCMV-Myc-gH in COS-7 cells through IFA. Recombinant plasmids pCMV-Myc-gH and pCMV-Flag-gL were co-transfected into COS-7, and identified the expressions through IFA. **a** Identification of expressions of pCMV-Flag-gL through anti-gL mouse polyclonal antibody; **b** Identification of expressions of pCMV-Myc-gH through anti-myc rabbit polyclonal antibody; Control: Incubated COS-7 cells as negative control

**Fig. 5:**
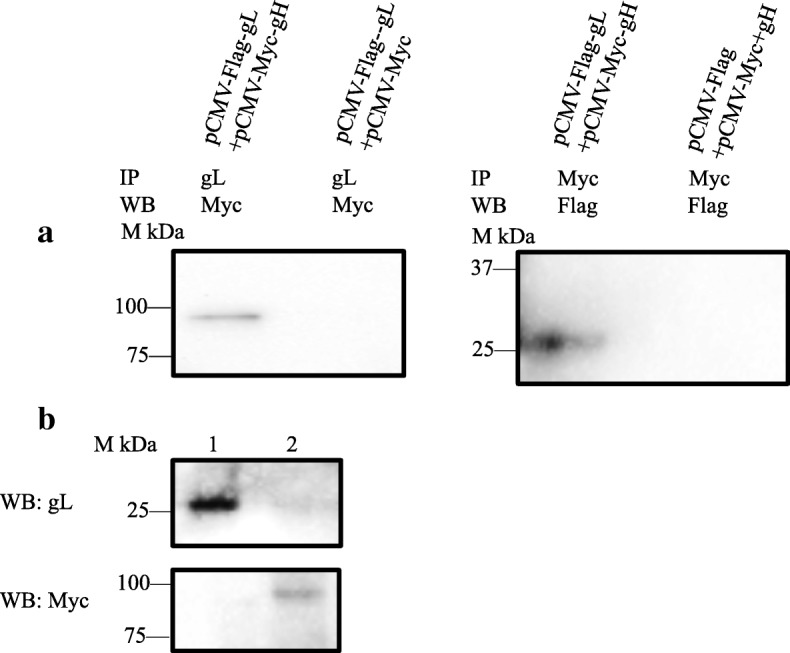
Co-immunoprecipitation of pCMV-Flag-gL and pCMV-Myc-gH. **a** COS-7 cells were co-transfected with experimental group (pCMV-Flag-gL + pCMV-Myc-gH) and control groups (pCMV-Flag-gL + pCMV-Myc or pCMV-Flag-gL + pCMV-Myc-gH) respectively. The uses of antibodies were as following: IP: anti-gL or anti-Myc polyclonal antibody for immunoprecipitation; WB: anti-Myc or anti-Flag polyclonal antibody for Western-blotting. **b** Recombinant plasmids pCMV-Flag-gL and pCMV-Myc-gH were co-transfected into COS-7 cells. Line 1: cellular extracts that analyzed by Western-blotting through anti-gL mouse polyclonal antibody; Line 2: Extracts that analyzed through anti-Myc rabbit polyclonal antibody

- On the basis of the co-transfection protocols above, recombinant plasmids pCMV-Myc-gH and pCMV-Flag-gL were co-transfected into COS-7 cells. The expressions of gH and gL after co-transfection was identified through IFA (Fig. 4). Compared to the negative control, green and red fluorescence was detected after incubation with either anti-gL or anti-Myc specific antibody (Fig. 4a, b), indicating the expression of gL and gH in COS-7 cells respectively. After co-transfection of experimental and control group 1 or 2 into COS-7 cells for 48 h, the glycoproteins in the experiment group pulled down by anti-gL antibody and detected by anti-Myc antibody with western-blotting, showed in a band around 93 kDa (gH) (Fig. 5a). Similarly, the glycoproteins in experiment group pulled down by anti-Myc antibody and detected by anti-gL antibody, showed in a band around 27 kDa (gL) (Fig. 5a). No visible band was observed in either of the control groups (Fig. 5a).

Further, the input of either gL or gH was tested by Western-blot. The results showed a band around 93 kDa (gH) and a band around 27 kDa (gL), confirming the expression of gH and gL respectively in COS-7 cells (Fig. 5b). Thus, both western-blotting results of gH and gL confirmed the interaction between them, a phenomenon seen in line with our expectations.

## Discussion

The interaction between glycoprotein H and L has been confirmed in HSV, HCMV and EBV, and gH/gL is conserved in the herpesvirus family [3, 7, 29]. Since DEV belongs to α-herpesvirus subfamily, the interaction of gH and gL was hypothesized in advance [3]. An analysis of the sequencings in GenBank and the prediction results generated using bioinformatics suggest that glycoprotein H is encoded by UL22 gene and has 834 amino acids (around 92.5 kDa) with one signal peptide and two transmembranes, while glycoprotein L, is encoded by UL1 gene, and has 234 amino acids (around 26.2 kDa) with one signal peptide but without a transmembrane region (data not shown).

An analysis of the similarities between DEV, HSV-2 and EBV was done by aligning the DNA sequence of both DEV gL and gH with either HSV-2 or EBV using the online alignment website (https://www.ebi.ac.uk/Tools/psa/emboss_needle/nucleotide.html). The alignment results showed that the percentage of similarity of DEV gH with HSV-2 and EBV is 39.4 and 34.4% respectively, while the percentage of similarity of DEV gL with HSV-2 and EBV is 42.6 and 33% respectively. It was been suggested that either gH or gL of DEV show some similarities with gH or gL of HSV-2 and EBV (data not shown). In addition, previous studies have demonstrated that the interaction regions of gH and gL in both HSV-2 (H1 domain) and EBV (Domain-1) are located in the N terminal of gH [8, 26]. Based on the analysis of both DNA similarity and protein construction, the location of the interaction domain of DEV gH and gL was hypothesized as being somewhere in the N-terminal of gH. However, further studies are needed to localized the interaction sites of the DEV heterodimer [12].

Fluorescence proteins can be used to study the intracellular co-localization between two different proteins [22, 24]. IFA is also a valid technique to analyze the expression and co-localization between two proteins at present [35]. However, compared to the fluorescence method used in this study, IFA is more complicated in that requires antibody responses. The detection of intracellular localization is significant for studying both the co-localization and also the interaction between two proteins, because proteins which may have interaction must attach to each other [30]. Based on the results of co-transfection of pEGFP-N-gH and pDsRED-N-gL into COS-7 cells in this study, a number of co-localization sites were detected with the fluorescence microscope. Therefore, the possibility of an interaction between gH and gL in cytoplasm is plausible based on finding of the partial co-localization within the cell. The results also show that gL essentially localizes in the cytoplasm after transfection for more than 12 h, and gH is expressed in both the cytoplasm and nucleus over the same period. Theoretically, either gH or gL belongs to membrane glycoprotein, which should play an important role in the surface of host cells [19]. In this study, however, a transfection of recombinant plasmids into mammalian cells, instead of the naturally viral infection (in vivo) was conducted. Thus, the co-localization of gH and gL might provide a clue about the interaction, but it is not enough to fully confirm the protein-protein interaction.

Co-IP is an available and useful approach to test the interaction between two different proteins currently [25]. Some research has proved the protein-protein interaction through Co-IP assay [15, 37], although the quality of specific antibodies are significantly essential [25]. Basically, massive cellular protein-protein interactions remained, after non-denaturing cell lysis. Once protein B interacts with protein A, protein B is be drawn to the side of the tube together with protein A by the Magnetic Racks, after the tethering of protein A with specific antibodies [13]. Thereafter, the existence of protein B with SDS-PAGE is tested, in order to confirm the interaction between protein A and B. In this paper, two experimental groups and two corresponding control groups were set respectively during the Co-IP assay. Contrary to the negative result in the control group, gH was detected when anti-gL antibody was used in Co-IP. Similarly, gL was detected when anti-Myc antibody (Myc-gH fusion protein) was used. Thus, detecting gH and gL respectively through SDS-PAGE, confirmed the interaction between gH and gL, on the basis of results of co-localization. The results from Co-IP are plausible because the bond between gH and gL is formed in a natural environment. It could be used to test not only the interaction between two or more proteins, but also the possible companion of specific receptors [13, 25].

In order to support the specific antibody during Co-IP experiment, the anti-gL mouse polyclonal antibody was prepared as a first step. Because gH protein could not be purified (data not shown), the Myc-gH tagged fusion protein was prepared for the subsequent test. Secondly, as both the gL and gH are membrane proteins of DEV, their interaction should occur on the cellular surface in vivo*.* However, instead of anti-gH antibody a Myc-tagged rabbit polyclonal antibody was used in the test, thus the Co-IP could not be conducted with both anti-gL and anti-gH specific antibodies. In other words, the interaction between gH and gL in natural state could not be tested. Although co-transfection of plasmids into mammalian cells could test the interaction of gH and gL as well, it neglects the possible scenario that the binding of gH and gL requires the intervention of a third protein [36], or the presence of some specific receptors [6, 16–18]. Based on the Co-IP results, however, the interaction was detected by co-transfection with two recombinant plasmids (in vitro), suggesting that gH can interact with gL without other proteins or specific receptors. But it would be better if both anti-gH and anti-gL specific antibody could be used in the experiment.

Moreover, considering the important function of membrane proteins such as gH, gL and gB during the entry of herpesviruses into host cells, the interactions among these proteins with binding their specific receptors are indispensable [11, 18, 21]. For example, in human herpesviruses, gH/gL can bind with gD, gp42, gQ or UL128–131 before the viral entry into host cells [1, 2, 24, 26]. During membrane fusion between HSV and host cells, gH/gL is initiated by receptor-binding gD at the first step, before activating the fusogenic potential of gB [16]. Also, glycoprotein D is found to be one of the essential membrane proteins in DEV, and has a similar function to that known in other herpesviruses [11]. Therefore, the hypothesis that DEV gD may interact with gH/gL during the entry into host cells could be accepted. Since gH and gL are essential proteins for DEV, it could be hypothesized that gH/gL would bind with other glycoproteins to promote DEV entry into host cells. A further study of the structure and function of gH/gL will be helpful to the study of protein-protein interaction in DEV, and also to the study of cell-cell fusion between herpesviruses and host cells.

## Conclusions

This research paper describes the co-localization of some of DEV gH and gL in COS-7, as well as the interaction between them through co-immunoprecipitation. The results provide an insight to the study of protein-protein interaction in DEV. In addition, because gH and gL are essential proteins for DEV, further study of the function or construction of gH/gL could be helpful not only to the study of membrane fusion, but also to the study of immunology and prevention of DEV.

## Abbreviations

Co-IP: Co-Immunoprecipitation
COS-7: African green monkey kidney cells
DEFs: Duck embryonic fibroblasts
DEV: Duck enteritis virus
DMEM: Dulbecco’s modified eagle’s medium
EBV: Epstein-Barr Virus
FBS: Fetal bovine serum
HSV: Herpes simplex virus
IFA: Indirect immunofluorescence assay
IPTG: Isopropyl-β-d-thiogalactoside
PBS: Phosphate buffered saline solution
SDS-PAGE: Sodium dodecyl sulfate polyacrylamide gel electrophoresis
TBST: Tris-buffered saline and tween 20
